# Genomic and phenotypic analyses of diverse non-clinical *Acinetobacter baumannii* strains reveals strain-specific virulence and resistance capacity

**DOI:** 10.1099/mgen.0.000765

**Published:** 2022-02-15

**Authors:** Mohammad Hamidian, Ram P. Maharjan, Daniel N. Farrugia, Natasha N. Delgado, Hue Dinh, Francesca L. Short, Xenia Kostoulias, Anton Y. Peleg, Ian T. Paulsen, Amy K. Cain

**Affiliations:** ^1^​ The iThree institute, University of Technology Sydney, Ultimo, NSW 2007, Australia; ^2^​ ARC Centre of Excellence in Synthetic Biology, Department of Molecular Sciences, Macquarie University, Sydney, NSW 2109, Australia; ^3^​ Infection & Immunity Program Monash Biomedicine Discovery Institute, Department of Microbiology, Monash University, Clayton, VIC 3800, Australia; ^4^​ Department of Infectious Diseases, The Alfred Hospital and Central Clinical School, Monash University, Melbourne, VIC, 3004, Australia

**Keywords:** *Acinetobacter baumannii*, resistance, niche adaptation, virulence, genome sequence, MLST, environmental, *Galleria mellonella*

## Abstract

*
Acinetobacter baumannii* is a critically important pathogen known for its widespread antibiotic resistance and ability to persist in hospital-associated environments. Whilst the majority of *A. baumannii* infections are hospital-acquired, infections from outside the hospital have been reported with high mortality. Despite this, little is known about the natural environmental reservoir(s) of *A. baumannii* and the virulence potential underlying non-clinical strains. Here, we report the complete genome sequences of six diverse strains isolated from environments such as river, soil, and industrial sites around the world. Phylogenetic analyses showed that four of these strains were unrelated to representative nosocomial strains and do not share a monophyletic origin, whereas two had sequence types belonging to the global clone lineages GC1 and GC2. Further, the majority of these strains harboured genes linked to virulence and stress protection in nosocomial strains. These genotypic properties correlated well with *in vitro* virulence phenotypic assays testing resistance to abiotic stresses, serum survival, and capsule formation. Virulence potential was confirmed *in vivo,* with most environmental strains able to effectively kill *Galleria mellonella* greater wax moth larvae. Using phenomic arrays and antibiotic resistance profiling, environmental and nosocomial strains were shown to have similar substrate utilisation patterns although environmental strains were distinctly more sensitive to antibiotics. Taken together, these features of environmental *A. baumannii* strains suggest the existence of a strain-specific distinct gene pools for niche specific adaptation. Furthermore, environmental strains appear to be equally virulent as contemporary nosocomial strains but remain largely antibiotic sensitive.

## Data Summary

1. *
Acinetobacter baumannii* genome sequences completed in this study, along with plasmids they carry, are deposited in GenBank and available under the BioProject PRJNA607827 and the following accession numbers: B9 (CP059548), 10_3 (CP059547), 10_4 (CP059546), E-011922 (CP059542), p3E-011922 (CP059543), p2E-011922 (CP059544), p1E-011922 (CP059545), E-072658 (CP061705), p1E-072658 (CP061713), p2E-072658 (CP061712), p3E-072658 (CP061711), p4E-072658 (CP061710), p5E-072658 (CP061709), p6E-072658 (CP061708), p7E-072658 (CP061707) and p8E-072658 (CP061706). Numbers in brackets indicate GenBank accession numbers. Names start with a ‘p’ followed by a strain name indicate individual plasmids found in each strain. All short read and long read data of these genomes have also been submitted to the sequence read archive (SRA) in GenBank and are available under the BioProject PRJNA607827 (https://www.ncbi.nlm.nih.gov/bioproject/PRJNA607827).
2. Eighteen publicly available *A. baumannii* genomes were downloaded from GenBank and used in phylogenetic analysis. These genomes include *A. baumannii* strains: A320 (CP032055), NIPH 1669 (KB849250), AB4456 (LREF00000000), DSM 30011 (JJOC02000000), ATCC 19606 (CP045110), LAC4 (CP018677) D4 (CP048849), D46 (CP048131), ATCC 17978 (CP012005), ACICU (CP031380), AB5075-UW (CP008706), AB0057 (CP001182), A1 (CP010781), AYE (CU459141), AB307-0294 (CP001172) D36 (CP012952), BAL062 (LT594096), D1279779 (CP003967) MDR-TJ, (CP003500), MDR-ZJ06 (CP001937), TCDC-AB0715 (CP002522) and *Acinetobacter pittii* strain PHEA-2 (CP002177), which was used as outgroup in phylogenetic analysis. Numbers in brackets indicate GenBank accession numbers.

**Impact Statement**

*
Acinetobacter baumannii* is a Gram-negative pathogen of critical importance because of its high incidence globally and its widespread resistance to multiple antibiotics. While the vast majority of *A. baumannii* infections are nosocomial, deadly infections outside the hospital are becoming more common. This study describes the genomics, phenotypes, and virulence potential of six *A. baumannii* strains isolated from diverse environments. The environmental strains displayed a distinctly antibiotic sensitive profile and lack of resistance genes compared to the clinical strains. Some environmental isolates clustered with the global clones GC1 and GC2 that cause the most nosocomial infections. The *Galleria mellonella* infection model was used to provide evidence that the environmental isolates were able to kill larvae *in vivo* at comparative rates to that of clinical *A. baumannii* strains. This study supports that environmental *A. baumannii* isolates have the potential to infect humans and, thus, they should be monitored a possible reservoir of infection.

## Introduction

*Acinetobacter baumannii* is a Gram-negative bacterium that has garnered increasing interest as a notorious opportunistic pathogen [1, 2], with its intrinsic [3] and laterally acquired [4] resistance to a wide range of clinically important antibiotics [5, 6]. In response to the global impact of multidrug resistant (MDR) *A. baumannii* infections, the World Health Organization has classified carbapenem resistant *A. baumannii* as a critical threat requiring the development of novel interventions [7]. In addition to resistance to multiple drugs, *A. baumannii* shows a robust ability to persist for months on a variety of clinical surfaces, which facilitates its transfer to new patients [8, 9].

*A. baumannii* is traditionally considered a nosocomial pathogen that causes hospital-acquired infections, including pneumonia, urinary tract and wound infections and bacteraemia, in critically ill or immunocompromised patients [1] yet rarely is reported to cause community-acquired infections. However, detection of community-acquired *A. baumannii* infections with high rates of morbidity and mortality in a number of countries [10–16] has prompted research on the virulence potential and molecular traits of non-clinical environmental strains. Unlike the majority of nosocomial strains, community-acquired infections tend to be more susceptible to antimicrobials [11, 17] and epidemiologically more closely related to strains recovered from environmental sources [11]. Although, the exact natural reservoir(s) of *A. baumannii* has not been identified [1, 18–20], this bacterium has been isolated from a range of non-clinical sources including meat and vegetables [21, 22], soils [22–24], anthropogenic surfaces [25], abattoir [26], various arthropods [27], estuaries [28] and agrarian sites [29]. *A. baumannii* has also been recovered from cattle and pig faecal samples [30]. Furthermore, recently, it has been suggested that *A. baumannii* is a One Health problem, which should be tackled by using a transdisciplinary approach, in which the health of humans, the environment, and animals are considered interconnected [31].

*A. baumannii* lacks well-defined host-specific virulence factors [32, 33] and its ability to infect hosts largely relies on its resistance to various abiotic stresses such as desiccation, acidity, oxidising agents, ability to pump out toxic chemicals via efflux mechanisms [33–38] and to activate certain metabolic pathways [39]. Considering that the majority of epidemic strains are multi-drug resistant [40–42], most studies of environmental *A. baumannii* strains generally focused on antimicrobial susceptibility and resistance traits. Thus, little is known regarding the genetic and phenotypic traits of environmental strains that directly facilitate infection ability and pathogenesis. Additionally, of the >8000 publicly available fully sequenced genomes, only <50 belong to strains collected outside of the hospital. These include a human body louse strain, SDF [43], and the strains from aerobic degradation (retting) of desert guayule shrubs NCIMB8209 and DSM30011 [44]. However, few comprehensive phylogenetic studies or detailed molecular analyses have been performed on *A. baumannii* environmental strains.

To investigate whether environmental strains possess intrinsic virulent traits we performed a side-by-side comparison of the genomes and virulence-associated phenotypic traits of six *A. baumannii* strains isolated from various non-clinical environments. We then put them into context with three well characterised clinical strains and one community-acquired strain from a bacteraemia infection of an indigenous Australian male [11] using a combination of *in vitro* and *in vivo* assays. We show that the environmental strains are phenotypically distinct from the clinical strains in terms both of antibiotic sensitivity and the ability to resist desiccation. However, they possess most of virulence traits found in the clinical strains such as resistance to human serum and shared similar catabolic capabilities. Most importantly, the majority of environmental *A. baumannii* strains were equally virulence as clinical counterparts in *G. mellonella* infection model.

## Methods

### Bacterial strains, species identification, culture conditions

All *A. baumannii* strains used in this study are listed in Table 1. *A. baumannii* strains 10–3 and 10–4 were isolated from Alabamian field soil demonstrating plant growth promotion and were kindly provided by John McInroy and Joseph Kloepper (Auburn University, AL, USA). *A. baumannii* B9 was incidentally isolated during sampling of the Seine River for multidrug resistant bacteria [45], and was provided by Patrice Nordmann (Paris-Sud University, Paris, France). *A. baumannii* E-011922 and E-072658 strains were procured from the VTT Culture Collection, Finland [46], and were previously isolated from kaolin and recycled fibre pulp Finnish paper mills, respectively. Ex003 was isolated in 2012 from the water sample of an artesian well in Koura, North Lebanon, in a previous study of non-clinical *A. baumannii* isolates in Lebanon [47]. All *A. baumannii* strains were routinely cultured in Luria Bertani (LB) broth at 37 °C.

**Table 1.** List of *A. baumannii* strains used in this study

Strain	Source	Classification	Country of isolation	Isolation year	Reference
10–3	Soil	Environmental	USA	Circa 1990	This study
10–4	Soil	Environmental	USA	Circa 1990	This study
B9	Seine River	Environmental	France	2010	[45]
E-011922	Paper mill kaolin	Environmental	Finland	2009	[46]
Ex003	Water	Environmental	Lebanon	2012	[47]
E-072658	Paper pulp mill	Environmental	Finland	2009	[46]
D1279779	Blood	Community-acquired	Australia	2009	[11]
AB5075-UW	Tibia/osteomyelitis	Nosocomial	USA	2008	[99]
BAL062	Bronchoalveolar lavage	Nosocomial	Vietnam	2014	[100]
ATCC17978	Meningitis patient	Nosocomial	USA	1951	[101]

### Whole genome sequencing and sequence analysis

Genomic DNA isolated from 10–3, 10–4, B9, E-11922 and E-072658 was isolated using the DNeasyTM UltraClean Microbial Kit (QiagenTM, Germantown, MD, USA) from cells grown overnight at 37 °C in LB inoculated from a single colony sequenced using Illumina MiSeq and Oxford Nanopore (MinION). Library preparation and barcoding for Illumina MiSeq and MinION (Oxford Nanopore Technologies, Oxford, UK) sequencing was performed by the UTS Core Sequencing Facility at the iThree Institute. Illumina sequencing generated paired-end short reads with approx. 50-fold coverage and an average length of 250 bp. MinION generated long reads with an average N50 of 18.2 kbp and 20–30-fold coverage. FastQC (v.0.11.9) (https://bioinformatics.babraham.ac.uk/projects/fastqc/) and Filtlong (v.0.2.0) (https://github.com/rrwick/Filtlong) were used (using default settings) to check the quality of Illumina reads and filter high-quality MinION reads, respectively. The high-quality Illumina and MinION reads were assembled *de novo* using a hybrid assembly approach with the Unicycler programme (v0.4.7). The CheckM software (available at https://github.com/Ecogenomics/CheckM) was used to examine the quality of genomes assembled here as it provides robust estimates of genome completeness and contamination [48].

Protein coding, rRNA and tRNA gene sequences were annotated using the NCBI Prokaryotic Genome Annotation Pipeline (v.4.11) [49]. The resistance genes, insertions sequences and polysaccharide loci were annotated manually using ResFinder (https://cge.cbs.dtu.dk/services/ResFinder/), ISFinder (https://www-is.biotoul.fr/), BLASTp (https://blast.ncbi.nlm.nih.gov/Blast.cgi?PAGE=Proteins) and Pfam (http://pfam.xfam.org/) searches, Kaptive was used to type the K and OC surface polysaccharides (https://kaptive-web.erc.monash.edu/) [50]. Sequence types were determined using the Institut Pasteur and Oxford MLST schemes (http://pubmlst.org/abaumannii/) from the genome sequence data.

### Phylogenetic analysis

A maximum likelihood phylogenetic tree was constructed from a core genome alignment of a set of *A. baumannii* strains representing known major clonal types, using short read data. The readSimulator programme (https://github.com/wanyuac/readSimulator) was used to generate short read data of genomes for which read data were not publicly available in GenBank. Briefly, the sequence reads for all isolates were mapped to the *A. baumannii* ATCC1797 strain, which was used as a reference (GenBank accession no. CP012004) using the Snippy software (v.2.0) (available at https://github.com/tseemann/snippy) to generate a whole-genome alignment. High-quality variant sites were called using SAMtools (v1.3.1.24) as described previously [51]. A maximum likelihood phylogenetic tree was inferred from the resulting alignment using RAxML (v.8) with the generalized time reversible (GTR) Gamma model of nucleotide substitution [52]. *Acinetobacter pittii* PHEA-2 (GenBank accession number CP002177.1) was used as the outgroup for the phylogenetic analysis. The phylogenetic tree along with additional features were visualised and plotted using ITOL (https://itol.embl.de/).

### Identification of prophage sequences

The PHASTER phage search database (http://phaster.ca) was used to find regions with significant identities to known phage genomes as described previously [53].

### Antibiotic resistance profile

Antibiotic resistance profile against 23 antibiotics was determined using the standard CDS (http://cdstest.net) disc diffusion method as previously described [54, 55]. Antibiotics tested were: AP, Ampicillin (25 µg); IPM, Imipenem (10 µg); MEM, Meropenem (10 µg); CAZ, Ceftazidime (30 µg); CTX, Ceftazidime (30 µg); SAM, Ampicillin/sulbactam (20/10 µg); CRO, Ceftriaxone (30 µg); SH, Spectinomycin (25 µg); S, Streptomycin (25 µg); CN, Gentamicin (10 µg), NET, Netilmicin (30 µg); AK, Amikacin (30 µg); K, Kanamycin (30 µg); N, Neomycin (30 µg); TOB, Tobramycin (30 µg); W, Trimethoprim (5 µg); RL, Sulfamethoxazole (100 µg); CIP, Ciprofloxacin (5 µg); NA, Nalidixic Acid (30 µg); RD, Rifampicin (30 µg), TE, Tetracycline (30 µg); FFC, Florfenicol (30 µg); C, Chloramphenicol (30 µg). Resistance and susceptibility were interpreted according to the Clinical and Laboratory Standards Institute (CLSI) guidelines for *Acinetobacter spp* [56]. and EUCAST [57] when a CLSI breakpoint for *Acinetobacter spp*. was not available.

### Desiccation assay

Exponentially growing cells (OD_600_=0.6) were harvested from 1 ml samples of LB cultures by centrifugation, and then were washed twice with 1 ml of sterile PBS and resuspended with MilliQ (MQ) water. MQ water was used to prevent additional osmotic stress during drying of the cell suspensions. Cell suspensions in MQ water were adjusted to an optical density at 600 nm (OD_600_) of 2.0, and then 10 µl droplets of each adjusted suspension were deposited onto a plastic (polystyrene) surface in 24-well sterile plates. The samples were allowed to dry for approximately 1 h in a biosafety cabinet at ambient temperature.

To estimate the survival time of *A. baumannii* cells, dried samples were incubated in a desiccator at ambient temperature in dark. The initial number of viable cells was determined by serially dilution of 10 µl culture in 1 ml PBS, followed by plating (100 µl) on LB plates in triplicate. To determine viability after drying, 100 µl of PBS was added onto each dried sample. The samples were rehydrated by incubating at room temperature for 10 min, mixed thoroughly by pipetting the suspensions up and down. The suspended suspensions were diluted serially in PBS, 10 µl of each dilution was then spotted onto LB agar plates, and the plate was incubated at 37 °C overnight. The viable cells on the dried surface were then inferred from the number of c.f.u. recovered from each dried sample. To determine the length of survival time of desiccated samples, three dried samples were sampled every 2–3 days for the first 12 days. Strains were considered no longer viable if no bacteria were recovered when undiluted samples were used straight on LB plates.

### Biofilm formation and capsule synthesis

For a biofilm formation assay we used the previously published method [58]. Briefly, overnight cultures were diluted 100-fold in 100 µl LB broth in a 96-well plate. Cells were then incubated for 24 h at 37 °C without shaking. The plates were washed three times with PBS to remove unattached cells and 125 µl of a 0.1 % crystal violet (CV) aqueous solution was added and incubated for 15 mins at room temperature. After rinsing three times with water and drying for 2 h, 125 µl of 30 % acetic acid in water was added to each well, incubated for 15 mins to allow complete solubilisation of CV and 125 µl of solubilised CV was transferred to a new flat bottom microtitre plate. Biofilm formation was then estimated by measuring absorbance in the plate at 550 nm using a 30 % acetic acid solution as the blank.

For qualitative estimation of capsule levels, we employed density gradient centrifugation as previously described [59], which is based on the effect of cell-associated capsule on bacterial density. Briefly, 1 ml of overnight grown cultures were centrifuged, washed with PBS and resuspended in 1 ml PBS. The OD_600_ of the cell suspensions was then adjusted to 1, translating to approximately 8×10^8^ cells ml^−1^, and 400 µl of the cell suspensions were loaded gently on the top of a solution of 37.5 % (AB5075-UW) or 47.5 % (ATCC 17978) Percoll in PBS. A second layer of 60 % Percoll was included to aid visualisation of the cells following centrifugation. The tubes containing biphasic Percoll solution and cell suspension were centrifuged for 5 mins at 3000 ***g***.

### Metal, osmotic, and oxidative stress assays

For all growth phenotypic assays, a single colony from Luria Bertani (LB) agar plates was used to inoculate 5 ml of LB broth medium. Overnight cultures were diluted to an optical density at 600 nm (OD_600_) of 0.01 in 105 µl LB broth with or without stress treatments in 96-well plates. We supplemented ZnSO_4_ (1.5 mM), CuSO_4_ (3 mM or 5 mM), H_2_O_2_ (0.5 mM) or 0.6 M NaCI in LB medium for zinc, copper, oxidative and osmotic stresses respectively. For all growth assays, cultures were incubated at 37 °C for 16 h with shaking at 200 r.p.m. in a PHERAstar FS Spectrophotometer (BMG Labtech). Cell growth was monitored at 0.1 h intervals by measuring OD_600_. Growth curves were used to calculate area under the curve (AUC) using Graphpad Prism 9.0. The difference in AUC between the strains was used as a proxy for fitness under the different stress conditions.

### Serum growth inhibition and serum bactericidal kill assay

For the serum growth inhibition assay, 10^5^ c.f.u. in 10 µl from exponentially growing cells in MH were transferred into 100 µl 50 % serum in MH plus 0.1 % tetrazolium dye in 96-well microplates. The plates were then incubated in an OmniLog reader (Biolog) aerobically at 37 °C for 48 h. Reduction of the tetrazolium-based dye (colourless) to formazan (violet) was monitored and recorded at 15 min intervals by an integrated charge-coupled device camera. The resultant data were analysed with the supplied manufacturer’s software. For the serum time kill assay, 1×10^7^ cells were incubated at 37 °C with phosphate buffer saline containing 40 % final concentration of serum or heat inactivated serum. Samples were taken every 30 min and the number of surving bacteria in each sample was determined by serial dilution and plating on LB agar plates.

### Virulence assay using the *Galleria mellonella* infection model

The *Galleria mellonella* infection experiments were performed as previously described in [60]. Briefly, triplicate assays of five larvae (200–230 mg) were injected with 1×10^7^ *A. baumannii* cells from the six environmental and four clinical strains separately. Survival and health of the larvae were enumerated at every day post-challenge for 6 days according to the *G. mellonella* Health Index Scoring System [61].

### Biolog phenotypic assay

The phenomes of *A. baumannii* strains were assayed with the Biolog GNIII Microplate system [62] to identify compounds that could serve as sole carbon sources (71 compounds). All phenotypic tests were performed as per the manufacturer’s protocol. Following inoculation, all PM plates were incubated in an OmniLog reader (Biolog) aerobically at 37 °C for 48 h. Reduction of the tetrazolium-based dye (colourless) to formazan (violet) was monitored and recorded at 15 min intervals by an integrated charge-coupled device camera. The resultant data were analysed with the supplied manufacturer’s software, resulting in a time-course curve for colorimetric change equating to respiration rate.

## Results

### Genomic features of environmental *A. baumannii* strains

The complete genome sequences of five environmental *A. baumannii* strains, 10–3, 10–4, B9, E-011922 and E-072658 from different environmental sources (Table 1) were determined using a hybrid MinION (Nanopore)-Illumina MiSeq approach, assembled using Unicycler (v0.4.8) resulting a single contig (complete chromosome) and various plasmids. We also included the previously reported genome of an environmental strains, Ex003, in our investigation [63]. General features of each genome were investigated and are summarized in Table 2. We first established that strains are indeed *A. baumannii *, by the presence of the intrinsic *oxaAb* oxacillinase gene, determining sequence types (STs), followed by performing phylogenetic analyses based on core genome alignment. We also included 11 known *A. baumannii* and 27 non-baumannii *Acinetobacter* species in our phylogenetic analysis for context. The six environmental strains clustered together with known *A. baumannii* strains (Fig. S1, available in the online version of this article). We also ran the CheckM on all six genomes of environmental *A. baumannii* to check the completeness and contamination [48]. We found 99.9–100 % completeness and with no to negligible contamination. On the basis of these observations, we concluded that these isolates are indeed *A. baumannii *. Moreover, two of the profiled strains, Ex003 (ST1) and B9 (ST2), were found to belong to global clone lineages 1 and 2 respectively, which are often associated with *A. baumannii* nosocomial outbreaks. The average chromosome size and GC content of these environmental strains was 3.78 Mb and 39 % respectively with E-072658, a strain from a paper pulp mill, the smallest at 3.68 Mb (Table 2). These sizes are slightly smaller but also consistent with the average values of 4.00 Mb and 39.03 %, respectively, for all the reported *A. baumannii* genomes in NCBI (https://www.ncbi.nlm.nih.gov/genome/?term=Acinetobacter+baumannii).

**Table 2.** General features of six environmental, one community and three nosocomial *A. baumannii* genomes

Strain	GenBank Acc. no.	Size (Mb)	No. of plasmids	GC (%)	Protein coding sequences (CDSs)	Insertion sequences (ISs)	tRNAs	ST*	OXA-Ab type
10–3	CP059547	3.81	0	38.9	3473	0	63	647	762
10–4	CP059546	3.82	0	38.9	3471	0	65	647	762
B9	CP059548	3.78	1	39.0	3447	82	63	2	66
E-011922	CP059542	3.97	3	39.1	3829	37	66	648	768
E-072658	CP061705	3.68	8	39.2	3310	440	63	649	562
Ex003	CP049314	3.94	1	39.0	3602	0	77	1	69
D1279779	CP003967	3.71	1	39.0	3388	18	65	267	180
ATCC 17978	CP012004	3.86	3	39.0	3527	14	72	437	256
AB5075-UW	CP008706	3.97	3	39.1	3771	8	74	1	69
BAL062	LT594095	4.04	1	39.1	3776	48	74	1550	66

ST* stands for sequence type based on *cpn60*, *fusA, gltA*, *pyrG*, *recA*, *rplB* and *rpoB*.

Subsequent analysis of the complete genomes of these environmental isolates, starting with identifying the presence of insertion sequences (ISs), revealed a variable number ISs present, ranging from zero in *A. baumannii* 10–3 and 10–4, to over four hundred ISs in E-072658, including members of the ISAba1 and IS4 family (Table 2). The complete lack of ISs was not limited to strains 10–3 and 10–4, as this has also been observed in an *A. baumannii* strain DSM30011, which was isolated in 1944 from resinous desert shrub guayule [64] and AB307-0294, an isolate from blood [65]. The relatively small genome size of E-072658 is likely due to extensive IS-mediated genome decay in a similar fashion to that observed in genome of *A. baumannii* SDF, an environmental strain obtained from human body louse which also harbours a large number of IS elements in its genome [66]. Similar IS-mediated genome remodelling with loss of some cellular functions has also been reported in another environmental *A. baumannii* strain (NCIMB8209), a companion strain of DSM30011 [44]. Although the role of IS*Aba*1 is unclear, it has been shown that IS*Aba*1 targets a specific position upstream of the intrinsic β-lactamase *ampC* gene of *A. baumannii* influencing expression of the *ampC* leading to high-level of resistance to β-lactams [67]. In spite of a smaller genome size, E-072658 is remarkable in that it carries eight plasmids in total (ranging in size from 4.4 kbp to 119 kbp), including the well-known six kbp plasmid pRAY [68] that carries the *aadB* tobramycin, gentamicin and kanamycin resistance gene cassette. To the best of our knowledge, this is the largest number of plasmids observed in a single *A. baumannii* strain. These results suggest a remarkable genetic plasticity in environmental *A. baumannii* strains.

### Phylogenetic analysis of environmental *A. baumannii* strains

**Fig. 1.**
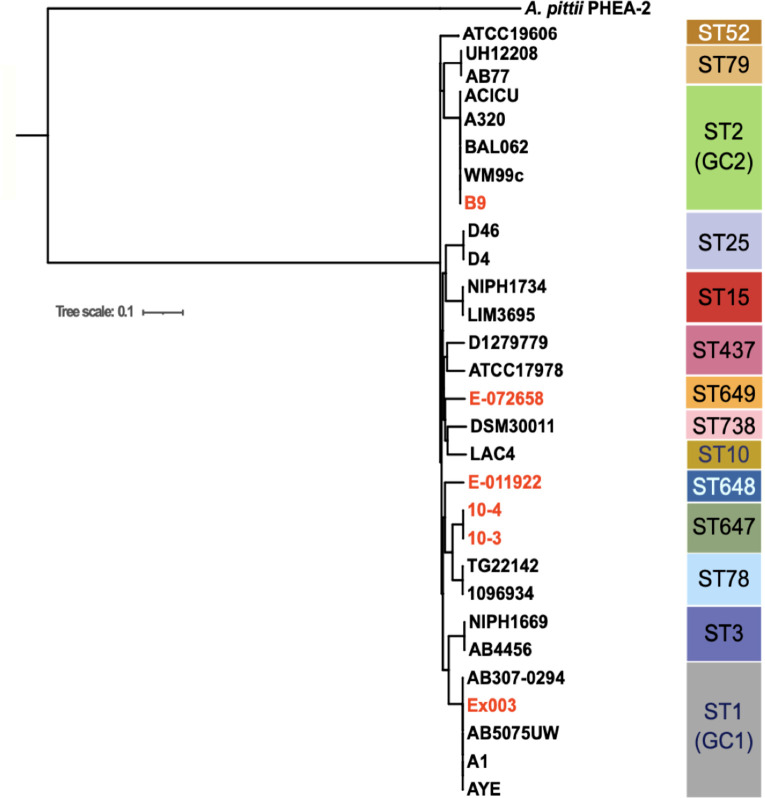
Phylogenetic relationship of environmental strains and representative community-acquired and nosocomial *A. baumannii* strains. A maximum likelihood phylogenetic tree was inferred from the resulting whole genome alignment using RAxML (v.8) with the generalized time reversible (GTR) Gamma model of nucleotide substitution [52]. *Acinetobacter pittii* PHEA-2 (GenBank accession number CP002177.1) was used as the outgroup for phylogenetic analysis. The phylogenetic tree along with additional features were visualised and plotted using ITOL (https://itol.embl.de/). See methods for detail. BAL062 belongs to ST1550, an SLV (Single Locus Variant) of ST2, which also belongs to global clone 2 (GC2).

The evolutionary relationship of the six sequenced environmental strains was inferred using the maximum likelihood phylogenetic analysis and put in context with fifteen completely sequenced strains of *A. baumannii *. As shown in Fig. 1, the environmental strains 10–3, 10–4 and E-011922 were most closely related to nosocomial *A. baumannii* strains belonging to ST78. The *A. baumannii* ST78 genotype first isolated in Italy [69] and subsequently spread worldwide [70]. The relationship of 10–3, 10–4 and E-011922 with nosocomial strains belonging to ST78 is interesting because the strains in this genotype were thought be monophyletic. Further, these environmental isolates are sensitive to most antibiotics whereas nosocomial strains belonging to ST78 display MDR phenotype [69, 70]. In contrast, B9, the Seine River strain [45], clustered with members of the global clone 2 (GC2) lineage, which is the most widespread clone of *A. baumannii* associated with multidrug nosocomial infections. Interestingly, E-072658, a strain from paper mill in Finland also closely related to the GC2 lineage. Similarly, Ex003, a strain from water (artesian well) in Lebanon clustered together with global clone 1 (GC1) nosocomial strains AB5075-UW. It is unclear whether nosocomial strains have found their way into the environment or nosocomial strains have environmental roots, nevertheless these results suggests that *A. baumannii* strains with potential to cause disease like others in their GC1 and GC2 clusters also can be found in environments.

### Genetic features important for virulence and persistence for environmental *A. baumannii*

To understand the variation in virulence amongst the clinical and environmental strains, we investigated genes that were previously identified as important for virulence in *A. baumannii *. Strains 10–3, 10–4 and Ex003 encoded a majority of the core virulence traits previously characterised in *A. baumannii *, while E-011922 strain did not harbour *galU*, which encodes UTP-glucose-1-phosphate uridylyltransferase, required for capsule biosynthesis (Table 3). Similarly, strain B9 lacked *wzb* and *bap* genes involved in capsule synthesis and biofilm formation, respectively. Interestingly, the genomes of *A. baumannii* E-072658 was lacking numerous core virulence genes, including those encoding outer membrane protein (*ompA*), pilin biogenesis and siderophore biosynthesis (Table 3). The absence of crucial virulence genes in the strain E-072658 might have been resulted from the IS-mediated niche specific adaptation as this strain harboured large number of ISs with reduced genome size.

**Table 3.** Distribution of genes involve in virulence in environmental and clinical (nosocomial and community-acquired) *A. baumannii* strains

Virulence determinant gene	locus_tag*	Environmental strains						Clinical strains			
		Ex003	B9	E-011922	10–4	10–3	E-072658	AB5075-UW	ATCC 17978	BAL062	D1279779
**Outer membrane Proteins**											
*blc*	ABUW_1048	+	+	+	+	+	+	+	+	+	+
*ompA*	ABUW_2571	+	+	+	+	+	−	+	+	+	+
*smpA*	ABUW_3034	+	+	+	+	+	+	+	+	+	+
**Biofilm Formation**											
*csuA*	ABUW_1488	+	+	+	+	+	−	+	+	+	+
*csuB*	ABUW_1489	+	+	+	+	+	−	+	+	+	+
*csuC*	ABUW_1490	+	+	+	+	+	−	+	+	+	+
*pgaA*	ABUW_1557	+	+	+	+	+	+	+	+	+	+
*pgaB*	ABUW_1558	+	+	+	+	+	+	+	+	+	+
*pgaC*	ABUW_1559	+	+	+	+	+	+	+	+	+	+
*pgaD*	ABUW_1560	+	+	+	+	+	+	+	+	+	+
*bap†*	ABUW_0885	+	+	+	+	+	+	+	+	+	−
*blp*	ABUW_0916	+	+	−	+	+	−	+	−	+	−
**Capsule Protein Biosynthesis**											
*ptk*	ABUW_3833	+	+	+	+	+	+	+	+	+	+
*wzb*	ABUW_3832	+	−	+	+	+	−	+	+	+	+
*galU*	ABUW_3819	+	+	−	+	+	+	+	+	+	+
**Siderophore**											
ABUW_2189	ABUW_2189	+	+	+	+	+	−	+	+	+	+
ABUW_2188	ABUW_2188	+	+	+	+	+	−	+	+	+	+
ABUW_2187	ABUW_2187	+	+	+	+	+	−	+	+	+	+
ABUW_2186	ABUW_2186	+	+	+	+	+	−	+	+	+	+
ABUW_2185	ABUW_2185	+	+	+	+	+	−	+	+	+	+
ABUW_2184	ABUW_2184	+	+	+	+	+	−	+	+	+	+
ABUW_2183	ABUW_2183	+	+	+	+	+	−	+	+	+	+
*bauA*	ABUW_1177	+	+	+	+	+	+	+	+	+	+
**Regulatory Proteins**											
*bmfR*	ABUW_3181	+	+	+	+	+	+	+	+	+	+
*bmfS*	ABUW_3180	+	+	+	+	+	+	+	+	+	+
*gigA*	ABUW_3260	+	+	+	+	+	+	+	+	+	+
*gacA/S*	ABUW_3504	+	+	+	+	+	+	+	+	+	+

*Locus tag is based on the whole genome sequence of *A. baumannii* strain AB5075-UW (GenBank: CP008706.1). ‘+’ stands for presence indicated gene(s) and ‘-’ stands for absence of gene(s).

†Organization and size of *bap* genes are shown in Fig. S2b.

### Environmental *A. baumannii* strains display diverse *in vivo* virulence potential

**Fig. 2.**
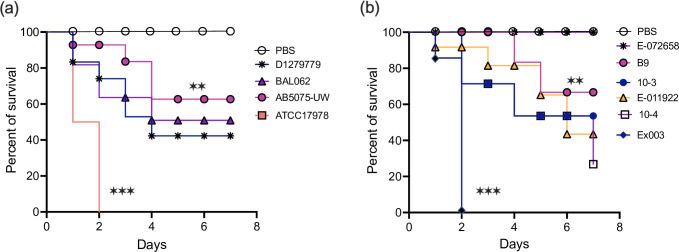
Virulence assays of environmental, community-acquired, and nosocomial *A. baumannii* strains using the *Galleria mellonella* infection model. Comparative survival analysis amongst *A. baumannii* nosocomial and community-acquired (**a**) and environmental (**b**) strains. Data are representative of three separate survival experiments, each performed with five larvae. Survival curves were constructed by the Kaplan-Meier method in GraphPad Prism. ns, not significant. **, *P*<0.01; ***, *P*<0.005.

*Galleria mellonella* larvae are an ethical animal model and effective for assaying the virulence capacity of bacterial pathogens, including *A. baumannii* [71, 72]. Thus, we employed this model to investigate the virulence of our six environmental *A. baumannii* strains. For comparison purposes we also included three nosocomial strains, ATCC17978, AB5075-UW and BAL062 and one community-acquired clinical strain, D1279779, in our investigation. As expected, we found that all four clinical strains exhibited virulence, albeit at a various degree of killing capacity (Fig. 2a) as reported previously [71]. More diverse virulence capacity was observed among the six environmental strains. The strain Ex003, belonging to the GC1 clonal complex, was observed to be the most virulent among the six environmental strains, whereas E-72658 did not kill any larvae at all (Fig. 2b). The virulence level of the other four environmental strains was comparable to that observed for AB5075-UW, BAL062 and D1279779. These results suggests that environmental strains can be as virulent as the clinical strains but not all have the capacity to kill larvae successfully.

### Establishing the characteristics required for stress resistance and long-term persistence

**Fig. 3.**
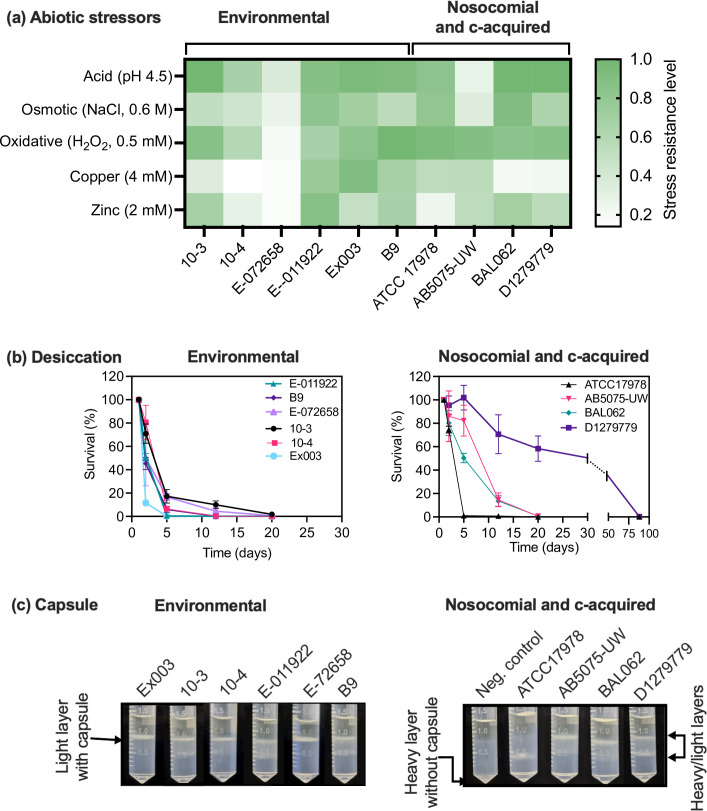
Characterization of phenotypic and virulence of properties of environmental, community-acquired (c-acquired) and nosocomial *A. baumannii* strains. (**a**) Stress resistance levels of *A. baumannii* strains was estimated by measuring the relative growth (area under growth the curve) in the presence of the indicated abiotic stressors. (**b**) Desiccation survival of *A. baumannii* strains. Overnight cultures grown in LB medium were sub-cultured and grown to mid-exponential phase. Cells were centrifuged, washed, and adjusted to an optical density at 600 nm (OD_600_) of 2.0 in MQ water, and then 10 µl droplets of each suspension was deposited onto a plastic (polystyrene) surface in 24-well. Surviving cells were enumerated at indicated time points after drying (see methods for detail). **c**) Density dependent qualitative analysis of capsule production in overnight grown cultures (see Methods for detail). The data are mean±SD of three independent experiments for stress and desiccation assays and one representative picture from two independent sensitivity assays for capsule production.

Most bacteria including *A. baumannii* often encounter stresses during host colonisation. Thus, in addition to virulence genes, the ability to resist environmental stressors also plays crucial role in successful colonisation in hosts and to persist in the environment over time [35, 73–75]. Therefore, we investigated the ability of the six environmental strains and four clinical strains to tolerate abiotic stressors such as acidity, salinity, oxidising agent and heavy metals (Fig. 3a). We found that both environmental and clinical (community-acquired and nosocomial) strains had a range of capacities to survive in stresses, with no one group consistently showing an obvious trend (Fig. 3a). The largest phenotypic differences within the environmental strains were observed between the two paper-mill strains, E-072658 and E-011922. In fact, E-072658 had the lowest stress resistance level as it was highly sensitive to almost all tested stresses (Fig. 3a), whereas E-011922 could resist all stresses tested to a relatively high degree.

Surprisingly, we observed differences in stress tolerance even within genetically similar strains. For instance, 10–3 and 10–4 were isolated from the same location (soil in Alabama) and of the same sequence type, yet stress resistance was significantly reduced in 10–4 compared to 10–3 which was generally more stress tolerant. Strain 10–4 was significantly more susceptible to copper stress than 10–3; the mean growth measured as area under growth curve (AUC) of the strain 10–4 was 8.28 [CI_95_ 8.17–8.30] compared to AUC of 16.36 [CI_95_ 16.29–16.43] under copper stress. Despite these differences, after closer examination of their genome sequences, only minor alterations of genomic composition were detected. The 10–4 strain found to be 6118 bp larger than 10–3, due to an internal duplication of an incomplete phage region (bases 1869068–1875197 of the 10–4 chromosome; GenBank accession number CP059546) encoding phage functions. Most *A. baumannii* genomes include 1–3 prophage regions (Hamidian; unpublished); however, analysis of this prophage region using the PHAge Search Tool (PHASTER) webserver [53, 76] showed that prophage in 10–3 and 10–4 is a novel one with 92 % DNA identity and 56 % coverage compared to its closest prophage found in the chromosome of *A. baumannii* strain K09-14 (GenBank accession number CP043953). In addition, 10–3 and 10–4 chromosomes also differed by 112 SNPs and/or small deletion/addition of 1–3 bases clustered in 21 chromosomal locations (Table S1). Most of these changes are in the two known biofilm associated genes, namely *bap1* and *bap2*, consistent with the observed significant difference in ability to form biofilm in these strains (Fig. S2). While a large number of changes were in intergenic regions, several others were in key genes including genes that could potentially influence the phenotypic traits (e.g. elongation factor Tu (locus_id H2784_16315) and RpoC; DNA-directed RNA polymerase subunit beta (locus id H2784_16270) (Table S1). Several changes were also found in genes encoding hypothetical proteins (locus ids H2784_17340, H2784_14365, H2784_00075, H2784_17335, H2784_03400 of the 10–3 chromosome). Stress tolerance differences observed in 10–3 and 10–4 could not be attributed to plasmid-borne genes as they do not contain any plasmids. The ability to form biofilm on the polystyrene plastic surface was also found to be highly diverse among the other environmental isolates presumably due to diversity in the *bap* and *blp* genes (Table 3, Fig. S2). Heterogeneity of *bap* and *blp* genes encoding biofilm associated proteins has also been demonstrated among *A. baumannii* strains [77].

We also compared the ability of both environmental and clinical strains to cope with desiccation stress over a period of 90 days. We found that *A. baumannii* strains displayed a range of capacities for desiccation tolerance, with Ex003, a strain belonging to ST1(GC1), being highly susceptible to dry conditions, as >99 % of the population died within the first 5 days (Fig. 3b). This finding was unexpected because most strains assigned to ST1 showed extremely high resistance to desiccation [42]. Similarly, the environmental strains E-011922, 10–3 and 10–4 belonging to ST648 and ST647 were also more sensitive to desiccation stress compared to most nosocomial strains (Fig. 3b). Interestingly, nosocomial strains assigned to ST78, the phylogenetically closest ST647 and ST648 were shown to be highly resistance to desiccation [42]. The other environmental strains also had a relatively low desiccation tolerance under our test condition, dying within 20 days and showing a drastic reduction in viability for all strains after just few days of desiccation. In contrast, the clinical strains were found to be better at initially coping with desiccation, but also became non-viable after 20 days. An exception for this was the widely used laboratory adapted reference strain ATCC 17978 which only survived 5 days (Fig. 3b). Among the ten strains used for study, the community-acquired strain D1279779 had by far the highest resistance to drying, surviving for an average of 88 days.

The presence of capsule is essential for *A. baumannii* pathogenicity, which not only plays a crucial role in evasion of host immune defences [78, 79], but is also necessary for both resistance to antimicrobial compounds and survival in serum. Additionally, it is required for survival in adverse environments, including stress arising from desiccation [78–80]. To investigate whether the environmental strains contain capsule, we performed a density-dependent gradient test on these strains, as well as the clinical strains for comparison [59]. As shown in Fig. 3(c), all environmental strains contained capsule, including the stress- and serum-sensitive strain E-072658.

It is worthwhile to note that the method used for capsule does not distinguish between capsular type. Previous studies have also demonstrated that there is no direct correlation between the amount or thickness of capsule and survival rate under desiccation [81]. It seems that the type and structure of CPS play important role in stress resistance including serum and desiccation [82]. We therefore investigated the distribution of surface polysaccharide loci using the KAPTIVE bioinformatic analysis [50]. As shown in Table 4, two of the environmental strains harboured novel capsular polysaccharide K-locus (KL) and outer core biosynthesis locus (OCL). Overall environmental strains harboured different OCL compared to nosocomial and community-acquired strains except for BAL062 (Table 4). Currently, the relationship between KL and/or OCL variation and resistance/virulence has yet to be fully elucidated. Although, the difference in OCL between nosocomial and environmental strains may contribute to the experimentally observed differences in desiccation tolerance.

**Table 4.** Distribution of surface polysaccharide loci

Strain	KL	OCL
10–3	155	1
10–4	155	1
B9	2	1
E-011922	239 (novel)	6 variant (novel)
E-072658	238 (novel)	13 variant (novel)
Ex003	1	1
D1279779	9	2
ATCC17978	3	2
AB5075-UW	25	2
BAL062	58	1

Gene loci involved in biosynthesis and export of capsular synthesis or K-locus (KL) and outer core biosynthesis OC locus (OCL) were determined using the KAPTIVE [50].

### Serum resistance in environmental *A. baumannii* isolates

**Fig. 4.**
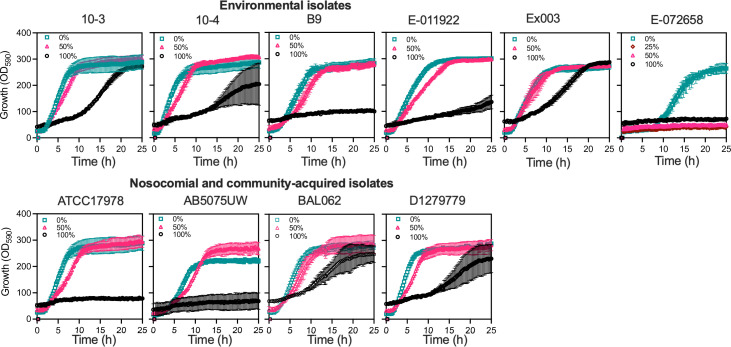
Serum resistance in environmental and nosocomial and community-acquired clinical *A. baumannii* strains. The growth inhibition of *Acinetobacter baumannii* in human serum. The bacteria (~1×10^5^ cells) were incubated with different concentrations of human serum in cation adjusted Mueller Hinton (MH) broth at 37 °C for 24 h in the presence of tetrazolium dye. The absorption of purple coloration of medium at 580 nm. Teal, indicates 0 % or MH control, while strawberry and (black) indicate 50 and 100 % (undiluted) human serum respectively.

It has been suggested that *A. baumannii* strains that cause bacteremia have increased resistance to human serum [83–85], including the complement system, which acts as an extracellular defence of innate immunity with antimicrobial activity [86]. To investigate whether environmental isolates are also resistant to human serum, we used the inhibition of respiration as a proxy of growth inhibition [11] for all six environmental isolates, as well as for the four clinical strains used as controls, in the presence of different concentrations of human serum. Of the six environmental strains, five were characterised as serum resistant, exhibiting little or no growth inhibition in the presence of 50 % serum in MH medium, and one strain, E-072658, was found to be highly sensitive to serum as its growth was completely inhibited by as little as 25 % serum (Fig. 4). We also found the serum growth inhibition results were entirely consistent with serum time kill assay (Fig. S3).

It is interesting that strains 10–3, 10–4, E-011922 and Ex003 were able to grow even in 100 % serum, suggesting that these bacteria have higher capacity to resist human serum. This is comparable to the growth of clinical strains BAL062 and D1279779, which could also grow in 100 % serum, while the other clinical strains could only grow in 50 % serum.

### Antibiotic resistance profiling

The genomes of environmental strains were investigated for the presence of genes known to be involved in antibiotic resistance. Additionally, the resistance phenotypic profile was also determined independently for each strain using a panel of 23 antibiotics via a disc diffusion method. We found that the environmental strains were largely sensitive to antibiotics and contained relatively few resistance genes (Table S2), although they harboured a majority of the core efflux proteins (e.g. AdeA, AdeFGH, AceI) known to be involved in the intrinsic multidrug resistance of nosocomial *A. baumannii* [87–89]. Strains 10–3, 10–4, E-072658 and Ex003 also lacked one or more genes encoding multiple drug transporters, including *adeABC*, and *abeM* (Table S2). However, E-072658 harboured the *sul2* gene required for resistance to sulphonamide. These strains also lacked the *pmrCAB* gene cluster involved in lipid A biosynthesis, the loss of which has been linked to increased resistance to polymxyin antimicrobials [90]. Antimicrobial resistance genes known to be carried within or activated by mobile genetic elements were absent from all sequenced non-nosocomial strains, except for the Seine River strain *A. baumannii* B9, which encoded a tetracycline efflux protein (*tetA*), an IS*Aba*1-activated chromosomal cephalosporinase (*ampC*), an oxacillinase (*oxa23*) and an aminoglycoside 3’-phosphotransferase (*strAB*). This organism also had Ser81→Leu and Ser84→Leu codon substitutions in DNA gyrase encoded by the *gyrA* gene and topoisomerase IV encoded by the *parC* gene, respectively. Both substitutions are known to contribute to fluroquinolone resistance [91].

**Fig. 5.**
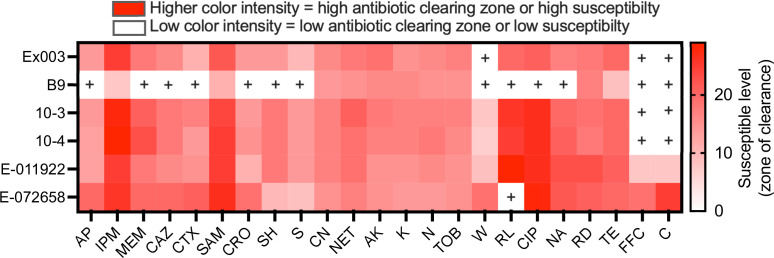
Heatmap showing antibiotic resistance and susceptible levels of six environmental *A. baumannii* strains. Antimicrobial susceptibility testing was performed on the strains according to the method established by the CLSI (formerly NCCLS) and interpreted with criteria published in 2005 [56]. Strains were tested using cation-adjusted Mueller-Hinton agar (BBL MH II; Becton Dickinson Microbiology Systems, Sparks, MD). AP, Ampicillin (25 µg); IPM, Imipenem (10 µg); MEM, Meropenem (10 µg); CAZ, Ceftazidime (30 µg); CTX, Ceftazidime (30 µg); SAM, Ampicillin/sulbactam (20/10 µg); CRO, Ceftriaxone (30 µg); SH, Spectinomycin (25 µg); S, Streptomycin (25 µg); CN, Gentamicin (10 µg), NET, Netilmicin (30 µg); AK, Amikacin (30 µg); K, Kanamycin (30 µg); N, Neomycin (30 µg); TOB, Tobramycin (30 µg); W, Trimethoprim (5 µg); RL, Sulfamethoxazole (100 µg); CIP, Ciprofloxacin (5 µg); NA, Nalidixic Acid (30 µg); RD, Rifampicin (30 µg), TE, Tetracycline (30 µg); FFC, Florfenicol (30 µg); C, Chloramphenicol (30 µg). The intensity of red colour indicates the level of susceptibility based on diameter of clearing or growth inhibition zone in mm (Table S4). Squares with white colour marked with ‘+’ stands for presence of known resistant determinant with no zone of clearance; see Table S2 for detail.

The presence or absence of antibiotic resistant genes was entirely consistent with the resistance phenotype, showing all environmental strains, except B9, were susceptible to most antibiotics tested (Fig. 5). The strain E-072658 was resistant to sulfamethoxazole as it harbours the *sul2* gene required for resistance to this drug [92]. Interestingly, all of the environmental isolates except for E-072658 were moderately or fully resistant to trimethoprim, despite lacking the trimethoprim resistance gene *dfrA,* which encodes a trimethoprim-insensitive homologue of the sensitive dihydrofolate reductase encoded by *folA* [93, 94]. Four isolates (Ex003, B9, 10–3 and 10–4) were resistant to florfenicol and chloramphenicol, most likely due to the presence of genes encoding efflux-mediated resistance mechanisms, including major facilitator super family (MFS) transporters [95, 96]. Given that *A. baumannii* B9 was isolated during screening of multidrug resistant bacteria in the Seine [45], and the subsequent revelation of it being an GC2 strain, the presence of laterally acquired genes involved in antimicrobial resistance suggests that this strain is likely derived from a clinical waste stream. The origin of this strain, however, has not been confirmed. The site the strain was isolated from was not in proximity to any hospital or wastewater treatment sites [45]. By employing the definition of MDR for *A. baumannii* [40] i.e. non-susceptible to ≥1 agent in ≥3 antimicrobial categories, we found only B9 as a MDR strain among the six environmental isolates.

### Metabolic phenotype of environmental *A. baumannii* strains

**Fig. 6.**
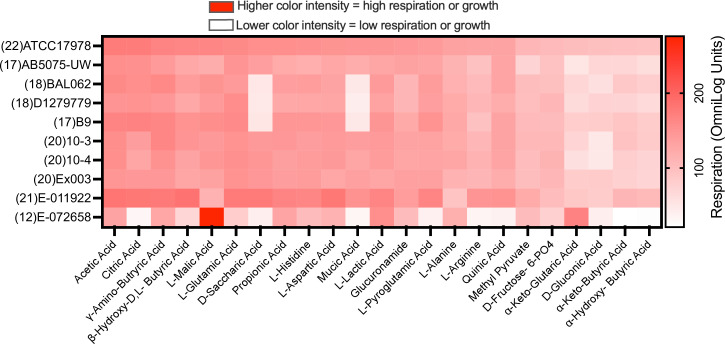
Heatmap showing relative respiration rates in environmental and community-acquired and nosocomial *A. baumannii* strains for different metabolic substrates. Level of respiration of six environmental and three nosocomial and one community-acquired clinical *A. baumannii* strains were determined using Biolog GN-III Microplates. The maximal growth height was expressed as OmniLog units. Phenotypes are arranged from strongest to weakest relative to *A. baumannii* ATCC 17978. The number in parentheses indicates the number of carbon source that the given *A. baumannii* strains can utilize out of 71 different sources in the GN-III Microplate.

Metabolic capacity is crucial for bacterial survival and infection, so we compared the metabolic phenotypic profile of six environmental *A. baumannii* strains and four clinical strains from different clonal complexes using Biolog phenotypic arrays. The Biolog GN-III Microplate is a respiration-based assay system that can test up to 95 different phenotypic traits simultaneously including the ability to metabolize 71 different carbon sources, representing all major classes of biochemicals such as sugars, hexose phosphates, amino acids, and carboxylic and fatty acids (Fig. 6, Table S3).

All ten *A *. *baumannii* strains tested shared a common trait that they cannot respire on hexoses (e.g. fructose, galactose, glucose) as a sole carbon source (Table S3). Of the 71 carbon sources tested they were able to utilize a combined total of 23; the majority of them being amino acids and a range of carboxylic acids (Fig. 6). We found that there was no clear indication that the environmental strains used different carbon sources to their clinical counterparts. The one exception was that E-072658 was able to use only 12 substrates as a sole carbon or nitrogen source (Fig. 6). The observed commonality in catabolic traits of the *A. baumannii* isolates from different environmental sources suggests that they mostly rely on their ability to catabolize amino and organic acids rather than hexose carbon sources.

## Discussion

The whole genome sequence of six environmental *A. baumannii* strains was completed and phylogenetic analysis indicated that four strains were epidemiologically and genotypically divergent from nosocomial representatives. However, the two river-associated *A. baumannii* strains, B9 and Ex003, were found to be closely related to the clinically significant global clones, GC2 and GC1 respectively. Interestingly, the strain B9 was highly antibiotic resistant and harbours the AbGRI1 resistance island, a hallmark of pathogenic MDR *A. baumannii* strains that belong to GC2. However, Ex003 was sensitive to most commonly used antibiotics, and is consistent with the absence of an AbaR-type resistance island, usually found within the chromosomal *comM* gene, The true origin of these strains, especially B9, could be nosocomial that have been released into the environment, but isolation of clinically-related MDR *A. baumannii* strains from the environment have also been described previously [97]. In 2014, a multidrug-resistant strain of *A. baumannii* was isolated within the paleosol of an abandoned quarry in Croatia and was genetically and phenotypically similar to a Croatian nosocomial strain of the GC1 lineage [97]. Recently, MDR *A. baumannii* strains have been isolated from streams close to an abattoir in South Africa [26].

With a wider lens, the comparative genomics of the various environmental strains and nosocomial *A. baumannii* show that a similar core genome of *A. baumannii* is still maintained [38]. The two fundamental differences that separate the genomes of environmental strains and clinical strains are genome size and the genes encoded in their respective accessory genomes. Typically, the genomes of environmental strains appear to be smaller than those of nosocomial strains, resultant from either considerable gene loss, as in the case of E-072658, or an absence of mobile genetic elements, such as in 10–3 and 10–4 strains which differ by the presence of a prophage. The genes encoded within the accessory genomes of environmental strains are primarily devoted to the acquisition and metabolism of novel compounds, whilst those in nosocomial *A. baumannii* are primarily involved in antimicrobial resistance, including the AbaR (in GC1s) and AbGRI (AbGRI1-5 in GC2 strains) resistance islands. The genome of one environmental strain, the Finish paper pulp strain, *A. baumannii* E-072658, is an exception, as the genome of this strain is characterized by extensive genome decay and rearrangement, presumably mediated by ISs. The aberrant genome architecture of E-072658 had complicated the analysis of this strain, but nonetheless the genome architecture of E-072658 was not a solitary event as similar arrangement was also detected in the genome of SDF, a human louse strain [43]. The observed reduction of genome in the strain E-072658 and concomitant with the reduced stress protection, catabolic capacities, and lack of virulence factor genes such as *ompA* might have contributed to its diminished virulence capability. In *A. baumannii* OmpA plays a crucial role in virulence by protecting cells from serum killing and improving biofilm formation [84, 85, 98].

Further, the presence of genes encoding multidrug resistance efflux proteins (Table S2) and typical virulence factors (Table 3) associated with resistance and virulence, respectively, in nosocomial *A. baumannii *, may suggests that high phenotypic similarities would occur between these diverse nosocomial strains. Commonality between them was seen in relation to their virulence capability; strains from both sources were able to colonize and kill *G. mellonella*. However, phenotypic differences such as antibiotic resistance profile and ability to tolerate desiccation stress between clinical and environmental strains were also observed.

In conclusion, our comparative genomic and phenotypic characterization of clinical and environmental *A. baumannii* strains provided important information on the genomic content and diversity of this species at both genomic and phenotypic levels. Generally environmental strains have smaller genomes and are more sensitive to desiccation stress. The accessory genomes of environmental strains seem to be dedicated to niche specific metabolic adaptation. On the contrary, nosocomial clinical strains adaptation involved antibiotic resistance and survival against desiccation stress. Most importantly, both clinical and environmental strains appear to display similar levels of virulence and share a similar complement of virulence genes. This study outlines how environmental *A. baumannii* could not only be a gene reservoir for pathogenic nosocomial strains, but also that they themselves have the potential to become pathogens. Therefore, further studies monitoring and cataloguing environmental *A. baumannii* in molecular detail is an important step forward to fully understand the origins and virulence potential of this important global pathogen. Coincidently, a similar argument has been recently proposed [31].

## Supplementary Data

Supplementary material 1

Supplementary material 2
