# Supplementary material for: Genomic and phenotypic analyses of diverse non-clinical Acinetobacter baumannii strains reveals strain-specific virulence and resistance capacity
Source: Microb Genom. 2022 Feb 15;8(2):000765. doi: 10.1099/mgen.0.000765 (PMC8942024; doi:10.1099/mgen.0.000765)
Supplement: Supplementary material 1 [file mgen-8-0765-s001.pdf]

Supplementary figures

Figure S1

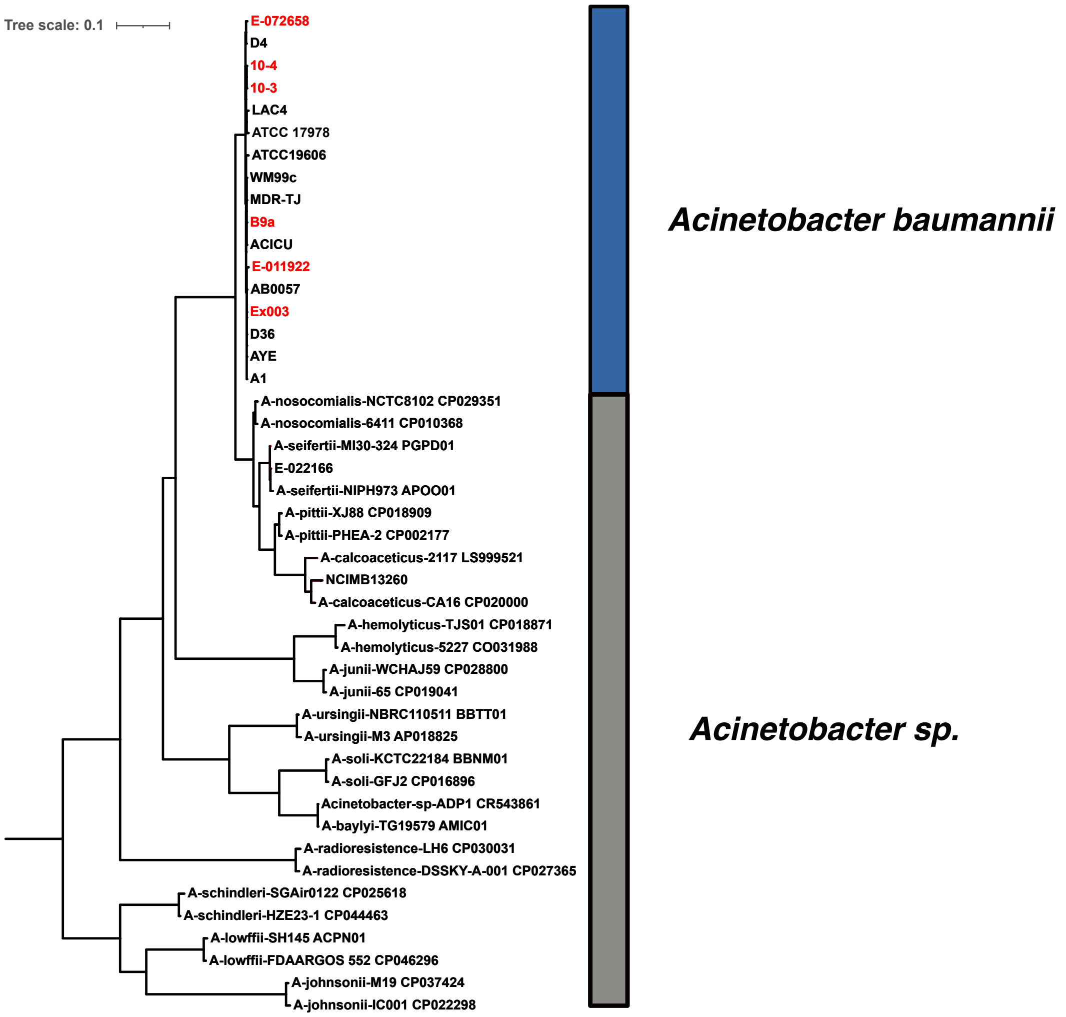

Figure S1. Phylogenetic analysis of six environmental *A. baumannii* strains in context to Acinetobacter species. The phylogeny was constructed using whole genome alignment of all genomes and inferred by RaxML software using GAMMA model. Numbers next to species names denote strain names and GenBank accession numbers, respectively. The tree was drawn and annotated in ITOL (https://itol.embl.de/). The tree scale is also shown.

Figure S2

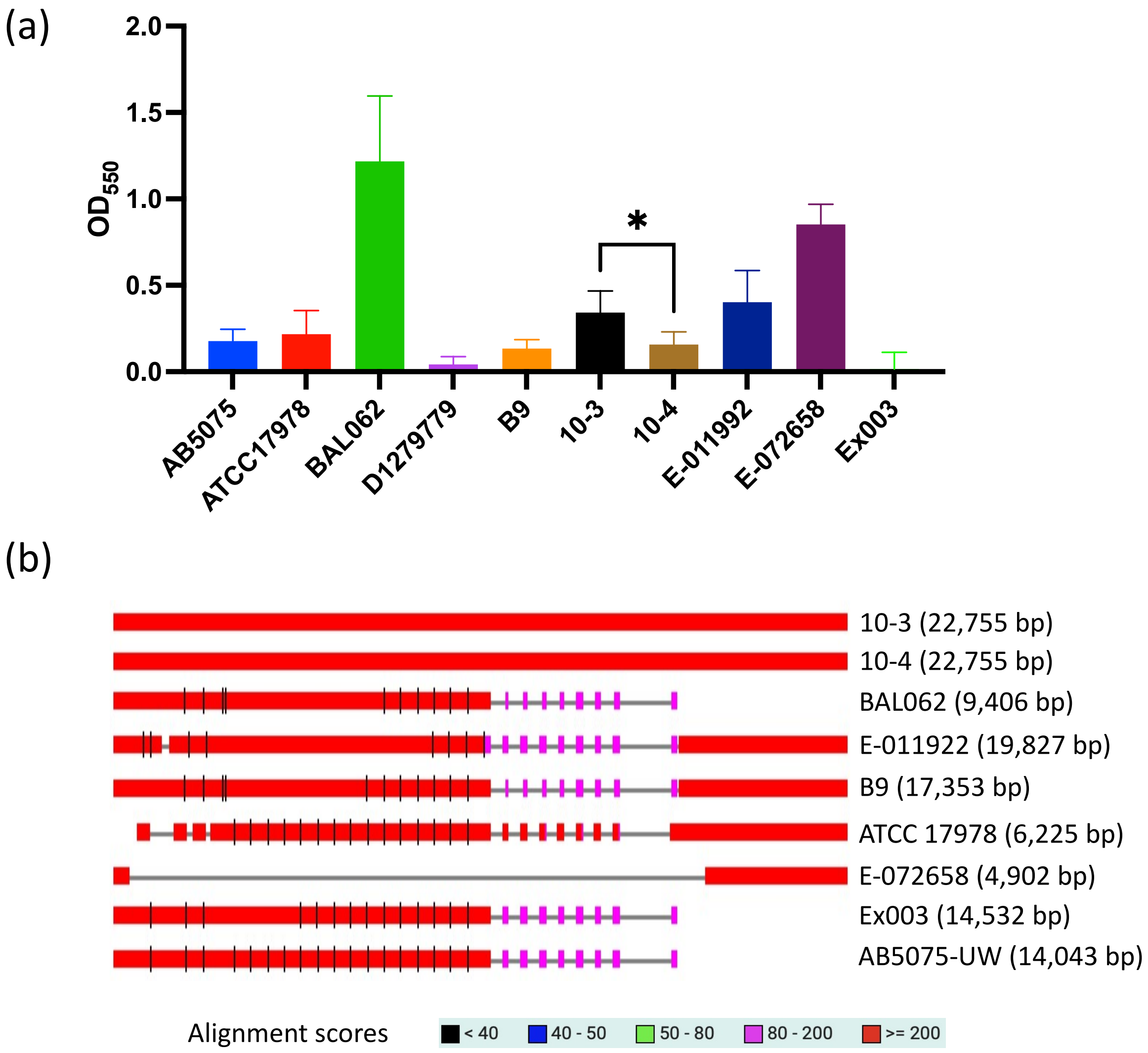

Figure S2. (a) Biofilm formation in environmental, nosocomial, and community-acquired *A. baumannii* strains. Bar graph showing estimates of crystal violet-based biofilm formation. The data represent the mean of at least two biological triplicates (±SEM). Statistical analyses were performed using a one-way ANOVA; * $p < 0.05$. (b) Organization and size of bap gene in six non-clinical and three clinical *A. baumannii* strains. *A. baumannii* strain D1279779 does not harbour *bap* genes. Numbers in parentheses are size in base pairs of *bap* gene region.

Figure S3

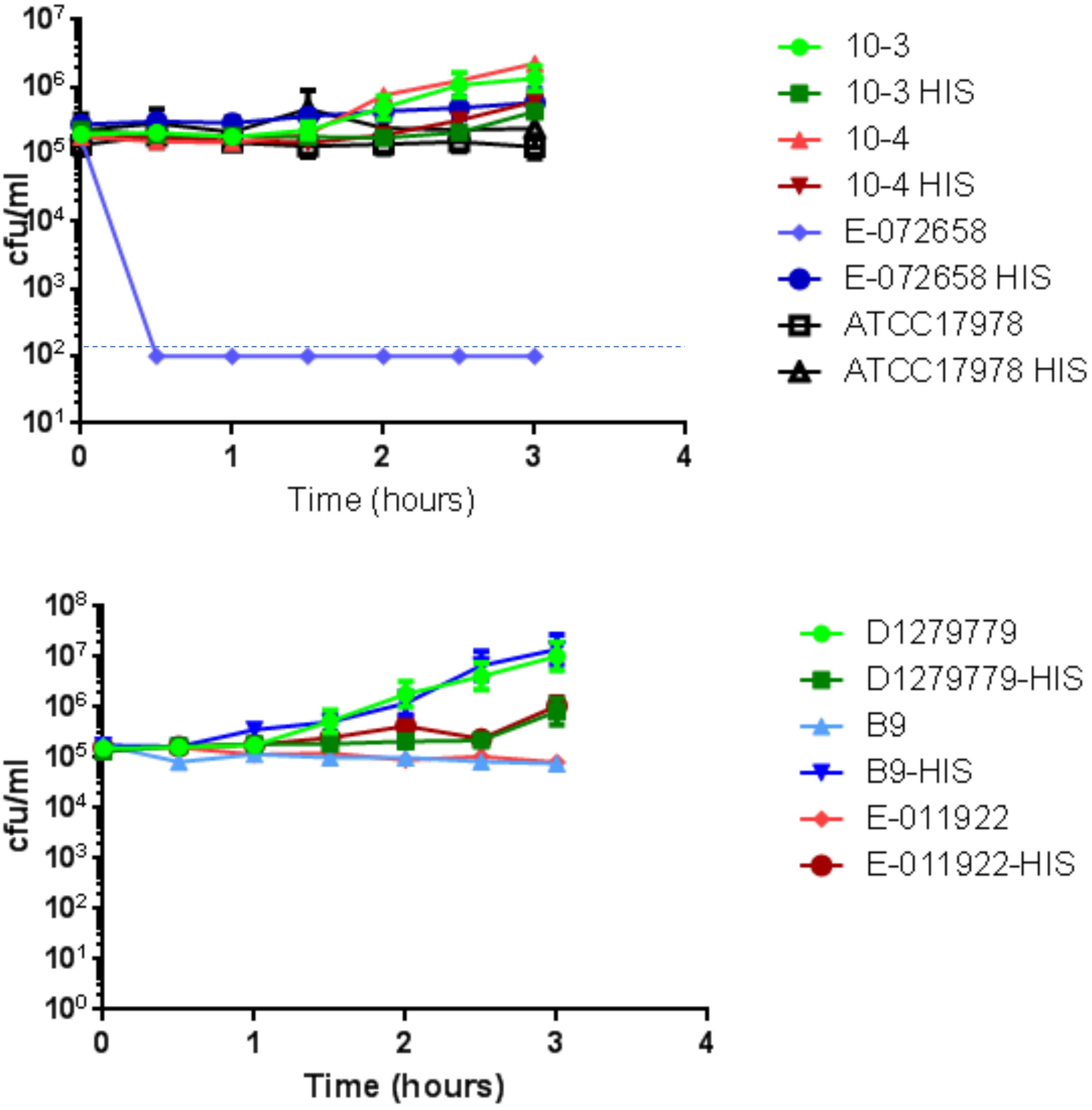
cfu/ml
Time (hours)
10-3
10-3 HIS
10-4
10-4 HIS
E-072658
E-072658 HIS
ATCC17978
ATCC17978 HIS
cfu/ml
Time (hours)
D1279779
D1279779-HIS
B9
B9-HIS
E-011922
E-011922-HIS

Figure S3. Serum resistance of *A. baumannii* strains. Strains were incubated in 40% normal Serum resistance of *A. baumannii* strains. Strains were incubated in 40% normal human serum or heat-inactivated serum (HIS), with colony forming unit (CFU) enumeration conducted at 30 min intervals over 3 hrs to measure survival. Each experiment was performed in biological triplicate with error bars representing at least two independent experiments. All strains, except for E-072658 were resistant to killing by human complement. Strain E-072658 was highly susceptible to killing by human complement with CFUs below the detection limit by 30 min. Dashed line indicates the detection limit of the assay. HIS means heat inactivated serum.
